# HSV-1-induced chemokine expression via IFI16-dependent and IFI16-independent pathways in human monocyte-derived macrophages

**DOI:** 10.1186/2042-4280-3-6

**Published:** 2012-10-14

**Authors:** Stine Søby, Rune R Laursen, Lars Østergaard, Jesper Melchjorsen

**Affiliations:** 1 Department of Infectious Diseases, Aarhus University Hospital, Brendstrupgaardsvej 100, Aarhus N, DK-8200, Denmark

**Keywords:** Herpes simplex virus, Innate, IFI16, Chemokine, PRR, DNA, Macrophages, Human

## Abstract

**Background:**

Innate recognition is essential in the antiviral response against infection by herpes simplex virus (HSV). Chemokines are important for control of HSV via recruitment of natural killer cells, T lymphocytes, and antigen-presenting cells. We previously found that early HSV-1-mediated chemokine responses are not dependent on TLR2 and TLR9 in human macrophages. Here, we investigated the role of the recently identified innate IFN-inducible DNA receptor IFI16 during HSV-1 infection in human macrophages.

**Methods:**

Peripheral blood mononuclear cells were purified from buffy coats and monocytes were differentiated to macrophages. Macrophages infected with HSV-1 were analyzed using siRNA-mediated knock-down of IFI16 by real-time PCR, ELISA, and Western blotting.

**Results:**

We determined that both CXCL10 and CCL3 are induced independent of HSV-1 replication. IFI16 mediates CCL3 mRNA accumulation during early HSV-1 infection. In contrast, CXCL10 was induced independently of IFI16.

**Conclusions:**

Our data provide the first evidence of HSV-1-induced innate immune responses via IFI16 in human primary macrophages. In addition, the data suggest that at least one additional unidentified receptor or innate sensing mechanism is involved in recognizing HSV-1 prior to viral replication.

## Introduction

Herpes simplex virus 1 (HSV-1) is a common human pathogen that primarily gives rise to orofacial and anogenital infections, which are controlled by the immune system in most cases. However, in newborns and immunocompromised individuals, the infection may become systemic and have a fatal outcome. Furthermore, HSV is a leading cause of virus-mediated encephalomeningitis [1,2]. Chemokines secreted at the site of infection are important for mobilization of effector cells to the site of infection [3-5]. In mice, production of CXC chemokine ligand 9 (CXCL9) and CXCL10 is important for controlling genital infection with HSV. These chemokines mediate recruitment of T lymphocytes and natural killer (NK) cells [6], which are essential for repression of HSV infection [7,8]. CC chemokine ligand 3 (CCL3) and CCL5 appear to be important regulators of anti-HSV immunity, as mice deficient in their receptor, CC chemokine receptor 5 (CCR5), are highly susceptible to genital HSV-infection [9]. The present study was designed to further evaluate chemokine expression and innate sensing during HSV-infection in human primary monocyte-derived macrophages.

Macrophages are sentinel cells in the innate immune response [10], and human primary macrophages are potent chemokine-producing cells important for controlling HSV infection [5,11,12]. Differentiated tissue macrophages are derived from monocytes, recruited from the blood. Macrophages display high phenotypic and functional diversity due to differentiation stimuli and the different environments in which they reside [10,13]. High numbers of primary macrophages are not easily purified from human tissues; therefore, most experiments with human primary macrophages are conducted using those differentiated from peripheral blood monocytes.

During the last decade, multiple pattern recognition receptors (PRRs) have been linked to innate recognition of HSV infection, which has been thoroughly reviewed elsewhere [14]. Briefly, cell surface Toll-like receptor 2 (TLR2) recognizes virion surface structures [15-17]; TLR3 recognizes virus-derived double-stranded RNA (dsRNA) in human fibroblasts [18]; TLR9 mediates sensing of HSV genomic DNA in endosomal compartments in human and murine plasmacytoid dendritic cells (pDCs) and murine macrophages [19-21]; and the RNA helicase melanoma differentiation-associated gene 5 (MDA-5) senses virus-derived RNA in the cytoplasm of human primary macrophages [22]. Finally, viral infection may be recognized by membrane fusion events [23]. In the present study, we investigated early innate recognition mechanisms in human primary macrophages with a focus on the recently discovered receptor for HSV-1 DNA, IFI16 [24] and fusion-mediated innate signals [23].

## Materials and methods

### Monocyte-derived macrophages

For macrophage differentiation, leukocyte-rich buffy coats were obtained from healthy blood donors (Skejby Hospital Blood Bank). Peripheral blood mononuclear cells were isolated and differentiated to macrophages, as previously described [22]. PBMCs were purified from freshly drawn blood from healthy donors. For all experiments approval was given from the local ethical committee and informed written consent from all participating subjects was obtained.

### Virus preparations

The KOS strain of HSV-1 was used in this study. The virus was produced as previously described [25]. Prior to use, the virus was thawed and used as replication competent virus or inactivate virus following exposure to UV light for 5 min [25].

### Small interfering RNA (siRNA) assays

After five days of cell culture in 6-well plates (Nunc, Roskilde, Denmark), macrophages were transfected with IFI16-specific siRNAs or control siRNA. Briefly, the cells were subjected to transfection using 100 nM of IFI16 siRNAs (Stealth RNAi mix; HSS105205, HSS105206, and HSS105207, Invitrogen, Glostrup Denmark) and recommended controls (Stealth RNAi control, Invitrogen, Glostrup, Denmark) combined with HiPerfect (Qiagen, Hilden, Germany) according to the manufacturer’s instructions. The cells were incubated with the HiPerfect-siRNA mix for 4 hours at 37°C before the media was changed to a fresh stock containing GM-CSF. Forty-eight hours after siRNA-transfections, the macrophages were used in infection experiments. Two hours before infection, the macrophages were supplied with fresh media without GM-CSF.

### Stimulation experiments

Macrophage experiments were conducted after 7 days of differentiation and PBMC experiments were conducted after overnight culture of the cells. Cells were treated with HSV-1 at a titer of 5 × 10^5^ pfu/ml (Strain KOS, MOI of 1). The TLR7/8-ligand R848 (InVivoGen, Toulouse, France) was used at a final concentration of 1 μg/ml. Type I IFN, IFN-α (R&D Systems, Abingdon, England) was used at the concentration 1000 U/ml. Lipofectamin2000 (invitrogen, Glostrup, DenmarK) was added to the cells to a final concentration of 5 μl/ml. Total RNA was harvested at 5 or 6 hours after infection using Trizol and purified according to the manufacturer’s description (Invitrogen, Glostrup, Denmark). Cell culture supernatants were stored at −80°C until ELISA analysis.

### Reverse transcription and real-time PCR

Total RNA was subjected to cDNA synthesis and subsequent real-time PCR analysis, as previously described [22,26]. For the siRNA experiments, the mean of control (untreated) siRNA treatments was used to quantify fold induction for the other samples. The gene-specific primers for IFI16, CCL3, CXCL10, and GAPDH used (Primers synthesized by DNA Technology, Aarhus, Denmark): IFI16 Forward, TAG GCC CAG CTG AGA GCC ATC C; IFI16 Reverse, TGA GGT CAC TCT GGG CAC TGT CTT; CCL3 Forward, ACT TCA GAA GGA CAC GGG C; CCL3 Reverse TGT AGC TGA AGC AGC AGG C; CXCL10 forward, AGG AAC CTC CAG TCT CAG CAC CA; CXCL10 Reverse, TGC TGA TGC AGG TAC AGC GTA CA; GAPDH forward, CGA CCA CTT TGT CAA GCT CA; GAPDH reverse, GGT GGT CCA GGG GTC TTA CT.

### ELISAs

CXCL10/IP-10 Cytoset ELISA kits (Invitrogen, Glostrup, Denmark) were according to the manufacturer’s protocol.

### Whole cell extracts and Western Blotting

After 6 hours of stimulation whole cell extracts were harvested essentially as described previously [26], the only change being that a commercially available cell lysis buffer was used (Biorad, Copenhagen, Denmark). To ensure equal loading of protein to the gels, protein was quantified using a DC protein assay according to manufacturer’s description (Biorad, Copenhagen, Denmark). For immunostains murine anti-IFI16 was used (clone 1GL, Santa Cruz Biotechnology, Santa Cruz, CA) at a concentration of 1 μg/ml in PBS and blots were visualized as previously described [26].

### Data analysis and statistics

Western blots were quantified using ImageJ. Statistical analysis was performed using Student t-tests.

## Results

Production of the chemokines CXCL10 and CCL3 during early HSV-1 infection occurs independently from viral replication

**Figure 1:**
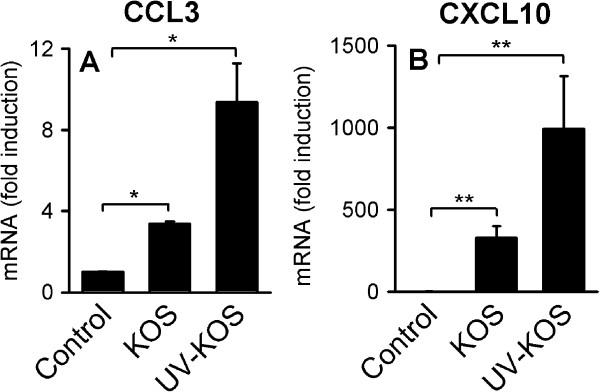
**Virus replication-independent expression of CCL3 and CXCL10 in HSV-1-infected human primary macrophages. **Human macrophages were infected with HSV-1 (KOS, MOI of 1) or equivalent amount of UV-inactivated virus. **A** and **B**) After 6 hours of infection total RNA was harvested and expression of CCL3 and CXCL10 evaluated by RT-qPCR. Data depicted represent mean +/− SD of three independent donors from one of two experiments showing similar results. *, p<0.05 and **, p<0.01 (*t*-test).

To evaluate whether early CCL3 and CXCL10 chemokine production is dependent on viral replication, we infected cells with replication-impaired UV-inactivated virus. UV-inactivated HSV-1 was fully capable of inducing CCL3 and CXCL10 expression (Figure 1A and B). Overall, the induction of CCL3 and CXCL10 was slightly elevated in cells treated with UV-inactivated virus compared with cells infected with replication-competent virus, indicating that viral replication products may inhibit CCL3 and CXCL10 expression. Thus, viral surface structures or intracellular components, such as viral DNA or tegument proteins, are directly recognized by human macrophages

### Expression of the innate DNA receptor IFI16 in human macrophages

**Figure 2:**
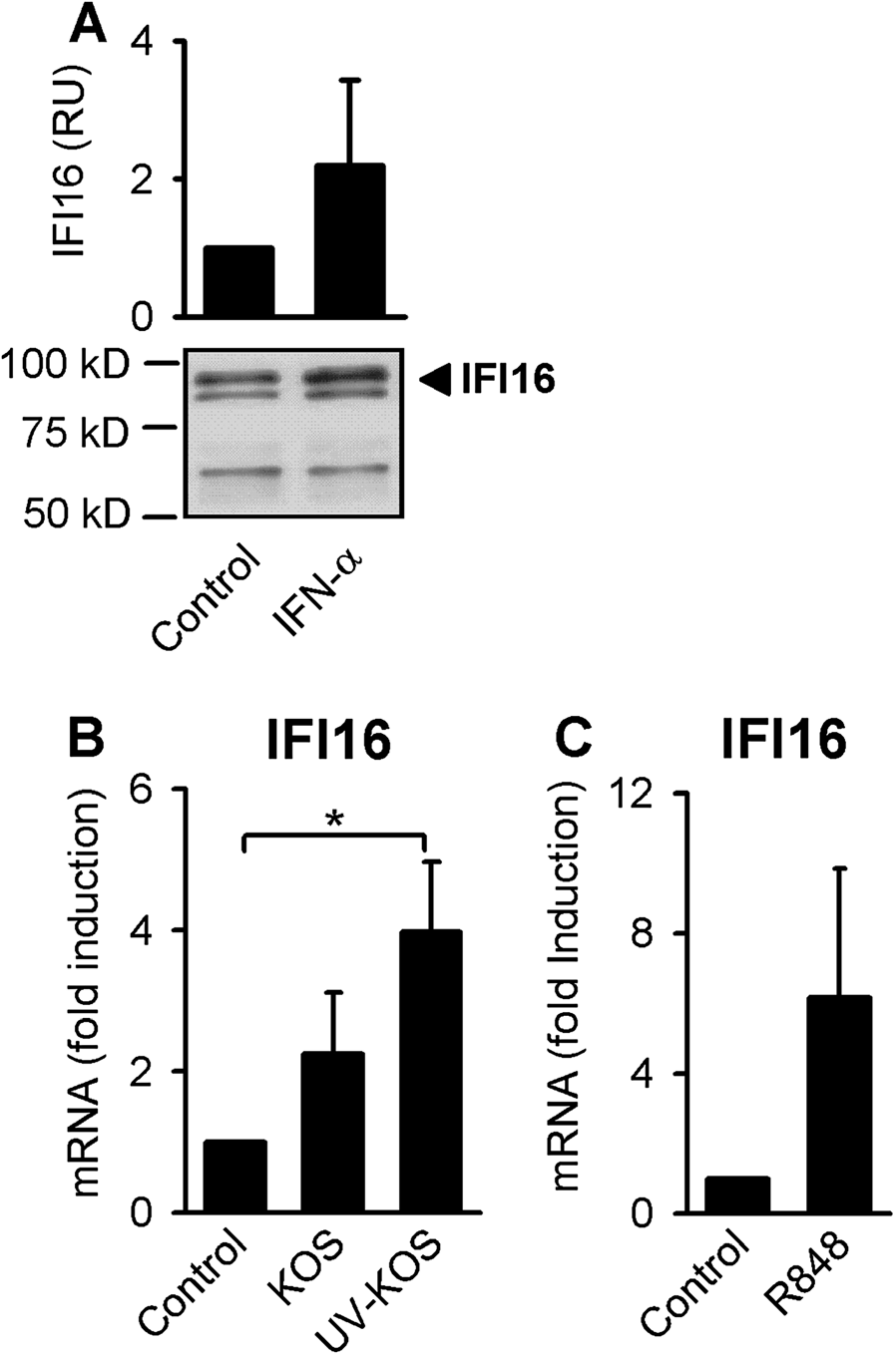
**IFI16 expression in human macrophages. A**) Macrophages were stimulated with IFN-α (1000 U/ml) for 6 hours and intracellular levels of IFI16 were assessed by Western blotting. **B** and **C**) Macrophages were stimulated with R848 (1 μg/ml) or infected with HSV-1 or UV-inactivated HSV-1 (KOS, MOI of 1) for 6 hours before assessing IFI16 mRNA-levels by RT-qPCR. The Western blot shown in A (lower panel) represents one of three donors showing similar results and the quantified data (upper panel) represents the mean +/−SD of the three donors from one experiment. The data depicted in B and C represents mean +/− SD of at least two donors of one of two independent experiments with similar results. *, p<0.05 (*t*-test).

We have previously shown that CXCL10 production is not dependent on TLR2 during HSV-1 infection and that human primary macrophages are unresponsive to TLR9 stimulation [22]. Therefore, we concentrated our studies on IFI16, an intracellular DNA receptor recently shown to mediate intracellular recognition of HSV-derived synthetic DNA in murine macrophages [24]. We found that IFI16 is present in human primary macrophages and that type I IFN induced IFI16 protein accumulation (Figure 2A). Looking at IFI16-induction caused by other stimuli, we found that early HSV-1 infection resulted only in a slight, approximately two-fold increase, in IFI16 mRNA levels; while, the induction mediated by UV-inactivated virus was roughly four-fold (Figure 2B). In addition, the TLR7/8-activating ligand R848 triggered a donor-dependent 2- to 8-fold increased accumulation of IFI16 mRNA (Figure 2C). Therefore, IFI16 is present and inducible in human primary macrophages.

### IFI16-dependent and IFI16-independent regulation of chemokine mRNA accumulation

**Figure 3:**
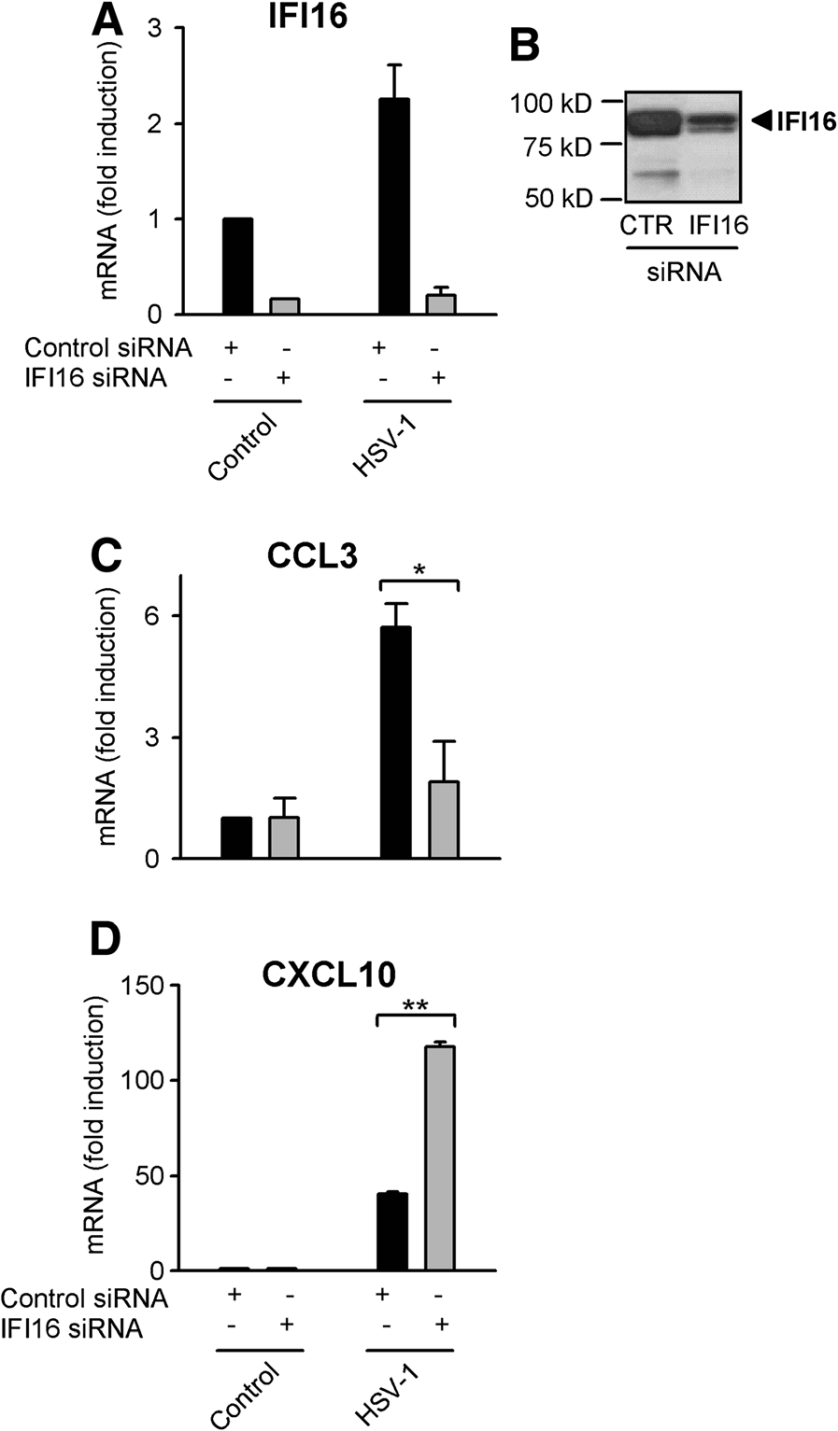
**Chemokine mRNA accumulation during HSV-1 infection via IFI16-dependent and IFI16-independent mechanism. **Macrophages were transfected with siRNA targeting IFI16 or control siRNA for 48 hours before infection with HSV-1 (KOS, MOI of 1) or stimulation with IFN-α (1000 U/ml). Mock infections were made as control. **A**) After 6 h, total RNA was harvested and IFI16 mRNA-levels were assayed using RT-qPCR. **B**) IFI16 protein-levels were evaluated by Western blotting in untreated macrophages 48 hours after transfection with control siRNA and IFI16 siRNA. **C** and **D**) Total RNA harvested at 6 hours post infection was analyzed for expression of CCL3 and CXCL10 using RT-qPCR. Results depicted represent one of four donors (A, C and D) and three donors (B) with similar results. *, p<0.05 and **, p<0.01 (*t*-test).

**Figure 4:**
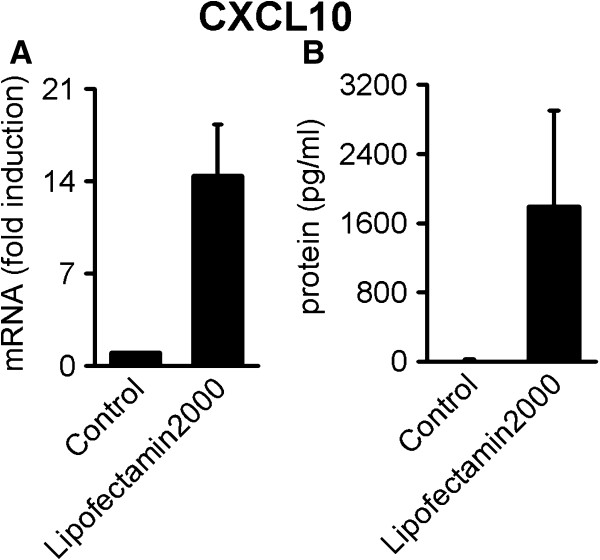
**Lipid-lipid interactions mediate CXCL10 induction in human primary cells. **PBMCs (**A**) and primary macrophages (**B**) were treated with lipofectamin2000 at the concentration 5 μl/ml and RNA was harvested at 5 hours and cell supernatants at 24 h. RNA was analyzed by qRT-PCR and supernatants analyzed for secreted CXCL10 using ELISA. The results in A and B represents mean+/− SD of two donors from one of two experiments showing similar results.

Using siRNA-mediated knock-down of IFI16, we assessed the production of CCL3 and CXCL10 in response to HSV-infection of human primary macrophages from two to three independent donors in three separate experiments. In all donors, there was 60 to 95% knock-down of IFI16 at the mRNA level (Figure 3A) and protein levels were also reduced (Figure 3B). As seen in Figure 3C, HSV-1-induced accumulation of CCL3 mRNA is dependent on IFI16. In contrast, IFI16 knock-down resulted in increased or unchanged CXCL10 expression during early HSV infection, suggesting that IFI16 is not required for CXCL10 expression (Figure 3D). The data suggest that CXCL10 is either induced via recognition of virion components, such as capsid or DNA, or via detection of the lipid membrane-coated virus entering the cell. The latter seems plausible, since we found CXCL10 was readily induced by lipid binding to both human PBMCs (Figure 4A) and human primary macrophages (Figure 4B). In contrast, CCL3 was not induced by Lipofectmine2000 in the macrophages (data not shown), suggesting that CCL3 mRNA accumulation is mediated via post-fusion steps*.*

Collectively, our data supports that the IFN-inducible DNA receptor IFI16 is present and inducible in human primary macrophages. Our experiments suggest that early CCL3 and CXCL10 induction occurs independently from virus replication, indicating direct recognition of virion structures or fusion events in human macrophages. Additionally, our data show that accumulation of CCL3 mRNA is dependent on the innate DNA receptor IFI16 during early HSV-1 infection; whereas, HSV-1 induction of CXCL10 proceeds via a yet uncharacterized pathway, possibly pertaining to viral fusion events.

## Discussion

Based on our findings here and previously [5,22], we suggest that HSV-1 is recognized by intracellular sensors present in the cytoplasm or the nucleus of human macrophages. Moreover, we hypothesize that virus fusion events result in expression of specific mediators of innate immunity. Our data support that IFI16 plays a role during early HSV-1 infection of human primary macrophages. HSV-1 DNA released during early infection may be recognized by IFI16, resulting in expression of CCL3 (Figure 3C); while, CXCL10 expression is mediated via an IFI16-independent pathway (Figure 3D). Furthermore, we found that IFI16 is upregulated by type I IFN and by the nuclear factor kappa B (NF-κB)-activating TLR7/8 agonist R848 (Figure 2A and C); yet, it is only slightly upregulated by HSV-1 during very early infection (Figure 2B).

UV-inactivated HSV-1 elicited a greater CCL3 and CXCL10 response compared to infection with replication competent virus (Figure 1A and B); therefore, the present data support previous findings suggesting that viral replication products inhibit IFN and chemokine expression in human macrophages as well as other cells [5]. For example, the viral protein ICP27 appears to harbor immune-modulatory capabilities, as demonstrated by more potent stimulation of CXCL10 and other cytokines in macrophages infected with a HSV-1 ICP27-deletion mutant compared with infection with wild type virus [5]. Evidence of immune-evasive activity has also been shown for several other HSV proteins, including ICP0, ICP34.5, Us11, vhs, and US3, which were reviewed previously [27,28]. IFN-inducible IFI16 was more effectively induced in macrophages infected with UV-inactivated HSV-1 compared with replication competent HSV, further supporting the notion that viral replication products counteract IFN responses.

The first cytoplasmic DNA receptor for HSV DNA was identified in 2007. The DNA-dependent activator of IFN regulatory factors (DAI) has been shown to mediate recognition of HSV DNA in a murine fibroblast cell line [29]. Since the initial publications relating to DAI, accumulating evidence has suggested additional DNA receptors exist. Within the past two years, major breakthroughs have been achieved, and an increasing number of DNA receptors have been associated with innate sensing of HSV, including the pyrin and HIN domain-containing protein (PYHIN) family member IFI16, which recognizes HSV-1-derived DNA in murine macrophages and Hela cells [24].

CCL3 mRNA accumulation was found to be dependent on IFI16 (Figure 3C). However, IFI16 knockdown did not consistently reduce CCL3 protein levels after 20 h of infection (data not shown). Reasons for this discrepancy between the mRNA and protein data include the following: i) CCL3 protein secretion may be transcriptionally regulated by factors other than IFI16 during HSV-1 infection in monocyte-derived macrophages or ii) CCL3 induction at the later time point is partly due to other virally induced factors, such as induced TNF-α [30], which we have previously found to be secreted 5 h after infection [5]. Therefore, studies evaluating CCL3 mRNA and protein accumulation at later stages during infection may be complicated by secretion of other mediators and by the accumulation of viral gene-products that selectively interfere with innate immune responses. However, the observed early IFI16-dependent CCL3 mRNA accumulation precedes the HSV-1-induced secretion of cytokines [5]. Therefore, we believe the CCL3 mRNA accumulation is mediated by direct sensing of HSV-1 virions.

Very recent data suggest a multifunctional role for IFI16 during HSV-1, HSV-2, and CMV infection. Studies from Gariano et al. demonstrated that siRNA knockdown of IFI16 enhanced viral replication [31], indicating IFI16 is a restriction factor for herpes virus replication. In theory, IFI16 could mediate innate recognition of HSV-1, while restricting replication at the same time. Thus, knock-down of IFI16 could enhance replication-dependent innate responses. Our data showed a two- to three-fold increased induction of CXCL10 in response to HSV-1-infection when macrophages were subjected to IFI16 knock-down (Figure 3D). Reduced IFI16 protein levels might result in decreased competition for viral DNA binding; thus, other DNA receptors able to stimulate CXCL10 expression have greater access to HSV-1 DNA not sequestered by IFI16. Another explanation could be redundant systems leading to CXCL10 mRNA accumulation: one dependent on virus replication (possibly being sensitive to IFI16 inhibition) and one via a pre-replication step. Further studies are necessary to determine whether IFI16 regulates mRNA accumulation and/or transcription or IFI16 acts as an innate receptor, and whether IFI16 restricts HSV-1 replication in human primary cells and/or sequesters viral DNA.

It should be noted that induction of CXCL10 may also be mediated by fusion or membrane-membrane interactions between the virus and cell. Collins et al. showed that HSV-1 replication is not required for induction of the IFN-stimulated genes CXCl10 and ISG56 [32], which is supported by our data showing replication-independent CXCL10 induction (Figure 1B). Moreover, a very recent study demonstrated that fusion events may indeed trigger innate responses, including expression of CXCL10 [23]. Accordingly, we found that membrane-membrane interactions induced CXCL10 in human primary macrophages and human peripheral blood mononuclear cells (Figure 4A and B). We hypothesize that viral fusion events could account for the observed replication- and IFI16-independent induction of CXCL10. Furthermore, we found that CCL3 is not induced by lipofectamin2000, suggesting that CCL3 mRNA accumulation is mediated via post-entry steps involving IFI16.

A number of other potential DNA sensors that recognize HSV-1 have been identified since this study was completed. The helicases DDX41, DDX60, DHX9, and DHX36 were identified as regulators of HSV-induced type I IFN and cytokine responses [33-35]. Zhang Z. et al. found that DDX41 mediates IFN-β, TNF-α, and IL-6 responses by recognizing viral DNA in the cytoplasm following HSV-1 infection in murine DCs [33]. Miyashita et al. found that DDX60 binds dsRNA and dsDNA in the cytoplasm and mediates induction of IFN-β and CXCL10 during HSV-1 infection in Hela cells; in addition, they found DDX60 promotes RIG-I and MDA-5 signaling [34]. Therefore, it remains to be determined whether DDX60 senses the HSV-1 genome or rather enhances the RLR-mediated signaling after HSV infection. Finally, Kim T. et al. found that DHX9 mediates TNF-α expression and DHX36 mediates IFN-α production in a human plasmacytoid cell line. In addition to the helicases, the DNA binding protein Ku70 was recently found to mediate type III IFN responses in HEK293 cells after DNA was introduced into the cells or cells were infected with HSV-2 [36].

Future investigations are necessary to further elucidate the role of IFI16 and determine the following: i) whether IFI16 functions as a primary innate receptor for HSV-1 DNA in human primary macrophages; ii) whether the aforementioned helicases DDX41, DDX60, DHX9 and DHX36, and Ku70 are sensors of viral DNA in human primary cells, including human primary macrophages; iii) which cellular responses these proteins regulate in primary cells; iv) whether HSV infection is sensed by DDX40, DDX41, DHX9, or DHX36 in human primary cells, including human primary macrophages; v) whether the specificity of DNA-induced innate responses is based on involvement of specific signaling adaptors and/or recognition of specific DNA sequences or lengths of DNA; vi) whether one of the newly identified DNA receptors mediates expression of CXCL10; vii) whether lipid-lipid interactions induce CXCL10/ISG expression in human primary macrophages during early HSV-1 infection or infection with other enveloped viruses; and viii) whether IFI16 is a restriction factor of HSV-1 infection in human primary cells.

Further details on the innate DNA receptors may provide important knowledge for the development of novel DNA vaccine adjuvants, specifically targeting receptors of interest. As proposed by A. Iwasaki, timed and targeted cytokine and chemokine responses may facilitate an influx of cells necessary for efficient vaccine responses in mucosal areas [37]. CXCL9 and CXCL10 play an important role in regulating NK cell and T lymphocyte influx to the site of infection and for control of vaginal HSV infection [6]; therefore, modulated production of specific chemokines may be imperative for future vaccine adjuvant candidates [37]. CCL3 could be important in terms of HSV clearance, as mice lacking the CCL3 receptor CCR5 are highly susceptible to vaginal HSV-2 infection [9]. In the context of vaccines, it is of interest that intracellular delivery of DNA forming a complex with lipids has yielded promising results in guinea pigs, decreasing viral replication and the severity of infection [38-40]. Based on the current knowledge, lipid-delivered DNA may provide two independent innate signals, one via lipid-interaction with the target cell membrane and one from the DNA targeting a cytoplasmic or nuclear DNA receptor.

Further investigation is necessary to determine the role of IFI16 and other DNA receptors in the development of novel DNA vaccine adjuvants targeting the cytoplasm. Furthermore, it remains to be determined whether DNA adjuvants will improve HSV vaccines in humans. However, a detailed understanding of cytoplasmic/nuclear DNA-host receptor interactions and the subsequent innate responses generated may provide rationale for use of delivering DNA to the cytoplasm or nucleus of cells as a vaccine adjuvant or direct antiviral therapy.

## Competing interests

The authors declare no conflicts of interests.

## Authors’ contributions

JM designed the study and lead the study. SS, RRL, and JM performed the experimental studies, analyzed the data, and made revisions. JM, SS, RRL, and LØ wrote the manuscript. SS and RRL contributed equally to the study. All authors approved the final version of the manuscript.
